# Acquired Zinc Deficiency in Preterm Infant Post-Surgery for Necrotizing Enterocolitis (NEC) on Prolonged Total Parenteral Nutrition (TPN)

**DOI:** 10.3390/pediatric16030046

**Published:** 2024-06-26

**Authors:** Mansour Al Qurashi, Hadeel Mohammad, Syed Sameer Aga, Ahmed Mustafa, Jubara Alallah, Mohammed Al Hindi, Mohammed Al Harbi, Mohammed Hasosah

**Affiliations:** 1 Department of Pediatrics, Ministry of National Guard Health Affairs (MNGHA), King Saud bin Abdul Aziz University for Health Sciences (KSAU-HS), King Abdulaziz Medical City, Jeddah 21423, Saudi Arabia; qurashim@ksau-hs.edu.sa (M.A.Q.); hadeelalhaj2018@yahoo.com (H.M.); asm.moustafa@gmail.com (A.M.); alallahjs@ngha.med.sa (J.A.); alhindimy1@yahoo.com (M.A.H.); harbimo01@ngha.med.sa (M.A.H.); hasosahmy@mngha.med.sa (M.H.); 2 King Abdullah International Medical Research Center (KAIMRC), King Saud bin Abdulaziz University for Health Sciences (KSAU-HS), Jeddah 21423, Saudi Arabia; 3 Basic Medical Sciences, College of Medicine, King Abdullah International Medical Research Center (KAIMRC), King Saud bin Abdulaziz University for Health Sciences (KSAU-HS), King Abdulaziz Medical City, Jeddah 21423, Saudi Arabia

**Keywords:** diseases, metalloenzymes, total parenteral nutrition (TPN), prematurity, alkaline phosphatase

## Abstract

Zinc (Zn) is a vital trace element that plays a pivotal role in protein synthesis, cellular growth, and differentiation and is involved as a cofactor of metalloenzymes, performing a wide variety of metabolic, immune, and synthesis roles. Zn is required at all stages of an infant’s and child’s development, and severe Zn deficiency has been reported to lead to slower physical, cognitive, and sexual growth. Preterm neonates are at a higher risk of developing zinc deficiency for a variety of reasons, including low Zn intake from enteral feeds containing breast milk, relative malabsorption due to immaturity of the gastrointestinal tract with limited absorptive capacity, increased urinary loss of zinc, and increased demand during the early developmental stages. Moreover, premature infants are at risk of gastrointestinal diseases like necrotizing enterocolitis (NEC), which can limit absorption capacity and potentially lead to malabsorption. TPN is frequently used in preterm infants to provide them with sufficient nutrients and calories. However, it has its own complications, including cholestasis, especially if used for prolonged periods. In this case report, we are presenting the case of a male preterm infant who was delivered by caesarean section at 26 weeks’ gestation. The baby developed an intestinal perforation due to NEC, for which he underwent surgery for resection of the necrotic bowel and the creation of a high ileal stoma and was put on prolonged total parenteral nutrition (TPN), which led to the development of zinc deficiency.

## 1. Introduction

Zinc (Zn) is one of the vital trace elements that is required for the efficient growth and development of several tissues as it plays a pivotal role in protein synthesis, cellular growth, and differentiation [1]. Zn plays an important role as a cofactor of metalloenzymes involved in metabolism, immune functions, protein folding, the development of the gastrointestinal tract, and gene expression [2,3]. Currently, around 1000 metalloenzymes and almost 2000 transcription factors are known to be dependent upon Zn for their catalytic activity [3,4,5,6]. Since Zn is required at all stages of a child’s development, severe Zn deficiency has been reported to lead to slower physical, cognitive, and sexual growth. It also manifests in skin disorders, immune compromise, and a higher frequency of acute illnesses in infants and children [1,7,8]. In the worst-case scenarios, Zn deficiency results in childhood stunting with an increased risk of morbidity as well as mortality in children [3,8,9].

**Figure 1:**
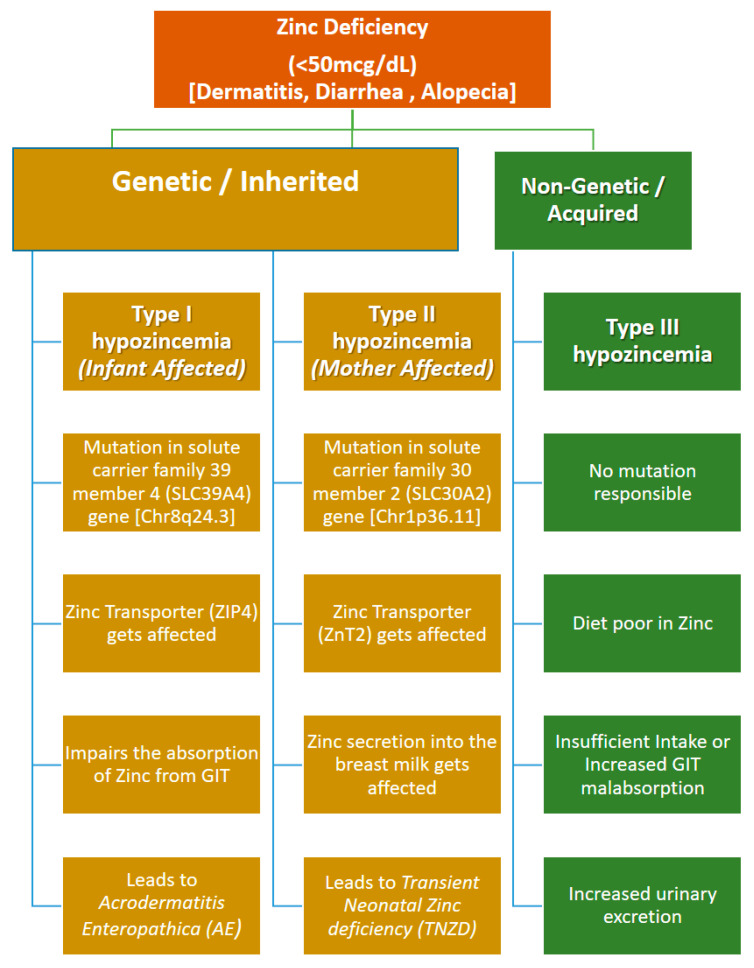
Different types of zinc deficiency and their etiology [8,11].

Zn deficiency may be either inherited or acquired depending upon the point at which Zn metabolism is being affected [7] (See Figure 1). Inherited Zn deficiency usually arises as secondary to the genetic mutations in the zinc transporter genes. Two of the important inherited Zn disorders have been characterized so far. One is due to mutation in the *SLC39A4* gene, which codes for zinc transporter ZIP4 in the affected individual and leads to impairment in the absorption of zinc from the gastrointestinal tract and hence low serum levels of the Zn (<50 mcg/dL); this is referred to as acrodermatitis enteropathica (AE) [7,10]. The second is due to mutation in the mother’s *SLC30A2* gene, which codes for the ZnT2 zinc transporter and leads to the reduced transport and secretion of zinc into breast milk; this disease is referred to as transient neonatal zinc deficiency (TNZD) [5,10,11]. The acquired etiologies of Zn deficiency are usually caused by a lack of a sufficient intake of zinc in the diet and may arise because of varied reasons, ranging from milk low in zinc levels, malnutrition, malabsorption, an increased loss of zinc, secondary gastrointestinal illness, increased urinary loss, and high demand [5].

Irrespective of the etiology of Zn deficiency, in its early stages all types exhibit the triad of clinical manifestations that include dermatitis, diarrhea, and alopecia [3,5,7,12]. Since, during fetal development, most of the Zn is acquired during the third trimester of the pregnancy, in preterm infants there is a high demand for Zn as a consequence of premature delivery [5,11,12]. Therefore, to avoid this potential risk, zinc is added to the Total Parenteral Nutrition (TPN) provided to premature infants in the neonatal intensive care unit (NICU) till full sufficient oral intake with adequate macro- and micronutrients is achieved [13,14]. After being absorbed in the small intestines via a carrier-mediated mechanism, Zn is taken up and consequently stored in four main organs of the body, the skeletal muscles (60%), bones (30%), liver (5%), and skin (5%). The skin usually contains about 60 µg/g of zinc in the epidermis and 40 µg/g in the upper dermis [4,5,6]. Zinc plays a crucial role in maintaining the structural integrity of skin by being directly involved in anti-inflammatory and wound-healing processes [3,4,5]; thus, skin lesions are common manifestations of Zn deficiency [4,12,14].

The provision of zinc supplementation is a standard intervention for all types of zinc deficiencies, which in the case of preterm newborns is provided via parenteral nutrition (PN) as they are unable to tolerate enteral feeds [12]. The goal of PN should be to provide elemental zinc up to a maximum of 5 mg/kg/day, which is enough to alleviate symptoms within a week and enable clinical improvement within six months of treatment [7,12,15]. Premature and low-birth-weight infants have been reported to develop a complication: PN-associated cholestasis (PNAC), nowadays referred to as intestinal failure-associated liver disease (IFALD), due to the prolonged use of PN (for more than 14 days) deficient in zinc and/or cysteine but rich in soy-based lipid emulsions (SOLEs) [16].

IFALD is one of the commonest serious complications of long term PN that results in liver injury due to intestinal failure (IF). It has complicated and multivariate causes and consists of a spectrum of liver illnesses, including cholestasis, biliary cirrhosis, and steatohepatitis [17,18,19]. SOLEs used for PN have a high phytosterol content, high ω-6 to ω-3 long-chain polyunsaturated fatty acid (PUFA) ratio, and very low levels of α-tocopherol. Phytosterols play a primary pathogenic role, as they are readily absorbed in the gastrointestinal tract, leading to their accumulation in the hepatocytes when given intravenously [20]. Fish-based lipid emulsions (FILEs) have high levels of α-tocopherol, low levels of phytosterols, and a significant concentration of anti-inflammatory ω-3 fatty acids like eicosapentaenoic acid (EPA) and docosahexaenoic acid (DHA), which have shown promising results in resolving cholestasis, reversing IFALD, and increasing survival [21,22].

## 2. Case Report

A baby boy was delivered by emergency caesarean section due to abnormal cardiotocography (CTG) at 26 weeks’ gestation to a gravida 5 para 2 mother who earlier had two abortions. The APGAR score was 4 and 8 at 1 and 5 min, respectively. The baby was intubated after birth and received surfactant therapy for respiratory distress syndrome (RDS) and his birth weight was 690 g.

Upon admission to a neonatal intensive care unit (NICU), the baby was sick, hypotensive, and required inotropic support (epinephrine). Echocardiography showed hemodynamically significant patent ductus arteriosus (PDA) for which he was treated with an acetaminophen course.

On day 6, the baby developed clinical and radiological signs of necrotizing enterocolitis, which was complicated by an intestinal perforation, underwent laparotomy, and was found to have multiple bowel perforations, for which a resection of the necrotic bowel and a high ileal stoma were created. There was difficulty in establishing enteral feeds due to significant feeding intolerance and a high stoma output, so the baby required total parental nutrition for a long period and, as a result, he developed cholestasis. His direct bilirubin reached up to 160 micromoles per liter, for which the baby started treatment on ursodeoxycholic acid, and trace elements were reduced to a twice-weekly dose instead of a daily dose in order to manage his worsening cholestasis status.

**Figure 2:**
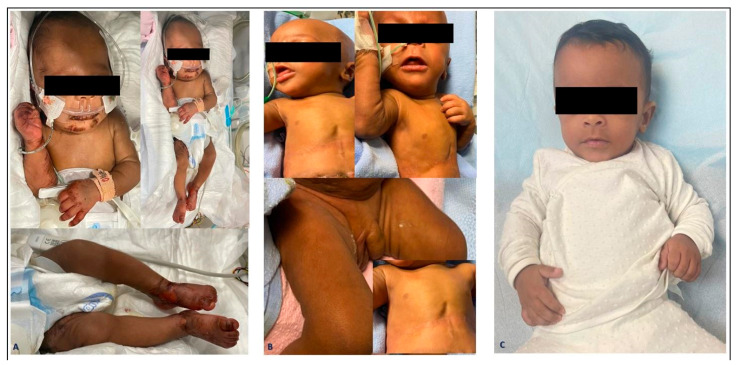
Typical skin lesions of zinc deficiency that include dry, scaly, and eczematous patches on the face (periorificial), anogenital areas, and extensor surfaces of the hands and feet (**A**), and significant improvement after zinc supplementation at 2 weeks (**B**), and before discharge (**C**).

**Figure 3:**
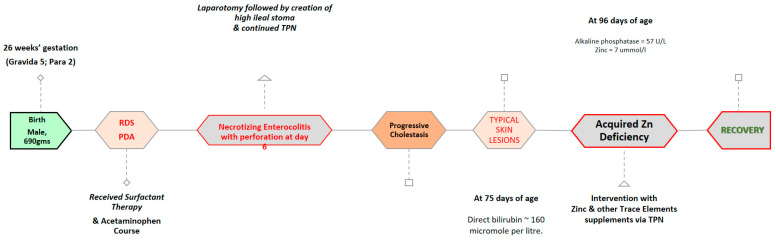
Timeline of the treatment of the patient and the consequent diagnosis of zinc deficiency.

At 96 days of age, he developed skin lesions/changes in the form of excoriation and redness on the face, abdomen, genitalia, and lower limbs (Figure 2 and Figure 3). His lab result at that time showed markedly relatively low alkaline phosphatase (a level of 57 U/L) and his baselines earlier ranged between 200 and 400 U/L, so zinc deficiency was suspected and serum zinc levels were requested, which were 1.7 ummol/L (normal range 9–10 ummol/L) (Table 1). The baby was restarted on oral zinc and resumed daily trace elements of TPN (Peditrace, Fresenius Kabi, Sweden). The recommended dose is 1 mL Peditrace/kg body weight/day (See Table 2 for composition). The repeated zinc level 5 days later was 8 and then 11 ummol/L. The skin changes described earlier had improved dramatically (Figure 2) and his alkaline phosphatase had risen to an acceptable level for a preterm infant.

## 3. Discussion

Total parenteral nutrition (TPN) is commonly used in preterm infants who are not able to receive enteral nutrition via an oral or enteral route [23]. TPN involves providing micro- and macronutrients including carbohydrates, lipids, amino acids, vitamins, trace elements, electrolytes, and water to the preterm infants (UVG, PICC) [23,24]. TPN, however, comes with its own complications—hyperglycemia, hypertriglyceridemia, and Hepatobiliary injury being topmost of them [23]. IFALD is a spectrum of diseases that can range from mild liver enzyme abnormalities to steatosis to eventual fibrosis or cirrhosis [16,23]. The European Society of Paediatric Gastroenterology, Hepatology, and Nutrition (ESPGHAN) advocates starting PN as soon as possible following surgery, as the early initiation of TPN with all the essential components may improve intestinal function, thereby reducing the risk of IFALD while enhancing nutrition and growth [25,26,27,28].

Here we report the case of a premature male who had to undergo an abdominal surgery for necrotizing enterocolitis and while on prolonged TPN developed cholestasis followed by the appearance of skin lesions/changes in the form of excoriation and redness. The diagnostic decision of an acquired zinc deficiency was made based on the low serum levels of zinc and clinical improvement following zinc supplementation. Similar cases have been reported previously, linking IFALD with the deficiency of dietary zinc, especially for infants born prematurely [3,7,16,29].

Zinc is one of the trace elements which cannot be stored in the human body, and hence a constant supply is to be sustained via diet to maintain its serum level around 700–1200 μg/L for viable functioning [29,30,31]. Poor nutrition, malabsorption, and loss of appetite has been linked to zinc deficiency in both adults and children [5,12,15]. Administration of zinc at doses of 5–10 mg/day is recommended to reduce the risk of zinc deficiency in children with a high demand for nutrients [12,29,32]. Zinc deficiency is more severe in premature infants for several reasons: firstly, zinc is accumulated efficiently only during the late gestation period; secondly, the fetus receives zinc from the mother only during the last ten weeks of gestation; and thirdly, preterm newborns have a high requirement for all trace elements, including zinc, to support rapid growth and development [3,12,16,33,34]. Additionally, human milk has higher zinc levels (>300 mcg/dL) only in the first few weeks of feeding and it gradually declines to <100 mcg/dL at 6 months after delivery [12,34,35].

## 4. Recommendations

The effective early supplementation of TPN as per the ESPGHAN guidelines [26] should be provided within 24 h of surgery if enteral feeding is not possible to prevent complications associated with this deficiency and improve a premature infant’s overall health. TPN should be administered in accordance with the nutrient requirements established based on age and weight, and it should include all necessary elements such as amino acids, carbs, fats, electrolytes, and vitamins. FILE-based TPN containing high quantities of antioxidants is recommended. Finally, an early transition to enteral feeding is advised to enhance gut health and speedy recovery. Ursodeoxycholic acid (UDCA) can be used to enhance bile flow and possibly alleviate symptoms in newborns with cholestasis.

## 5. Conclusions

This case highlights the importance of monitoring trace element levels in premature infants on long-term TPN, as well as the potential risk of acquired zinc deficiency in those with cholestasis, which may require adjustments of their prolonged provision of TPN. Close monitoring of TPN devoid of SOLEs but enriched with FILEs would help with the early resolution of complications. Effective supplementation as per the ESPGHAN guidelines can prevent complications associated with this deficiency and improve a premature infant’s overall health.

## Figures and Tables

**Table 1:** Biochemical profile of the case before and after the treatment.

Biochemical Variable	1 Month Before	2 Weeks Before	1 Week Before	Time of Appearance of Skin Rash	1 Week After	2 Weeks After	1 Month After
Direct Bilirubin (mmol/L)	75	104	161	-	112	142	137
Alkaline Phosphatase (U/L)	315	94	57	-	480	643	655
AST (U/L)	75	148	115	-	142	182	74
ALT (U/L)	45	178	124	-	126	109	96
GGT (U/L)	43	43	47	-	70	51	77
Zinc (mcg/dL)				1.71	8.06	11.66	-

**Table 2:** Trace element composition of PEDITRACE (pH of 2.0 and 38 mOsm/kg water).

	Compound	per mL
1	Zinc chloride	521 µg
2	Copper chloride (dihydrate)	53.7 µg
3	Manganese chloride (tetrahydrate)	3.60 µg
4	Potassium iodide	1.31 µg
5	Sodium fluoride	126 µg
6	Sodium selenate (anhydrous)	4.38 µg
7	Sodium	70 µg/mL
8	Potassium	0.31 µg/mL

Ref: https://www.fresenius-kabi.com/nz/documents/Peditrace_DataSheet.pdf (accessed on 1 January 2024).

## Data Availability

Data are contained within the article.
